# Novel diagnostic biomarkers associated with macrophage-microglia in spinal cord injury

**DOI:** 10.3389/fimmu.2025.1634014

**Published:** 2025-09-10

**Authors:** Manyi Zheng, Yunduo Jiang, Fangyu Liu, Yansong Wang

**Affiliations:** ^1^ The First Affiliated Hospital of Harbin Medical University, Harbin Medical University, Heilongjiang, Harbin, China; ^2^ Department of Orthopedic Surgery, The First Affiliated Hospital of Harbin Medical University, Harbin Medical University, Harbin, China; ^3^ The Key Laboratory of Myocardial Ischemia, Ministry of Education, Harbin Medical University, Harbin, China; ^4^ NHC Key Laboratory of Cell Transplantation, Harbin Medical University, Harbin, China; ^5^ Key Laboratory of Frigid Zone Cardiovascular Diseases, Harbin Medical University, Harbin, China

**Keywords:** spinal cord injury, single-cell RNA sequencing, bulk RNA sequencing, microglia, macrophage, inflammation, immune microenvironment

## Abstract

**Background:**

Spinal cord injury (SCI) is a devastating disorder featuring serious motor dysfunction and proprioceptive deficits due to central nervous system (CNS) damage. During its progression, macrophage-microglia (MM) cells are rapidly activated and play pivotal roles in the inflammatory response through various mechanisms. However, limited research has investigated the differential gene expression between the two groups before and after injury, and these important genes may therefore serve as potential biological diagnostic markers.

**Methods:**

Our study utilized the Gene Expression Omnibus (GEO) data for SCI-related MDEG identification. Key hub genes were screened using multiple machine learning(ML) algorithms. Their predictive potential was subsequently validated using independent datasets, and their association with immune cell infiltration was assessed. An *in vitro* model of SCI was established, and quantitative polymerase chain reaction (qPCR) experiments were conducted to verify our findings.

**Results:**

Single-cell RNA sequencing identified 16 distinct cellular subpopulations, among which MM cells were split into four subsets according to functional characteristics. Enrichment analyses, including KEGG, GO, and GSEA, revealed that the MDEGs were closely linked to key biological processes in SCI. From 200 genes associated with critical WGCNA modules, three hub genes, including EMP3, GNGT2, and SGPL1, were identified through four ML algorithms as differentially expressed before and after injury. Predictive models based on these genes demonstrated strong performance in both internal training and external validation cohorts. Preliminary analysis of immune infiltration and gene-immune cell correlation suggests an association with M2 macrophages. Furthermore, *in vitro* modeling of post-injury inflammation confirmed the elevated expression of EMP3, GNGT2, and SGPL1.

**Conclusion:**

Our study identifies EMP3, GNGT2, and SGPL1 as potential diagnostic biomarkers associated with MM cells in SCI. The foregoing findings lay a theoretical basis for elucidating their biological roles and formulating future treatment strategies.

## 1 Introduction

Spinal cord injury (SCI) is a devastating neurological disorder that severely impairs motor function and inflicts profound psychological trauma on affected individuals. SCI is most commonly precipitated by acute trauma to the spinal column, with primary injury mechanisms including vertebral fractures that embed bone fragments into the spinal canal, and vertebral dislocations that result in ligamentous disruption of the spine (1). This initial insult is frequently followed by secondary injury processes, which represent the body’s maladaptive attempts at repair. These secondary mechanisms involve hemorrhage, edema, reactive oxygen species (ROS) production, inflammatory cell infiltration, neuronal and glial cell death, and scar formation - all of which may exacerbate tissue damage (2). With the ongoing global demographic trends of population growth and aging, the incidence and prevalence of SCI, particularly those resulting from falls in the old population, are projected to rise (3). Current therapeutic strategies for SCI encompass pharmacologic interventions, surgical procedures, and rehabilitative approaches (4). Epidemiological data from the 1980s to the present reveal that the incidence of SCI is generally lower in developing countries than in developed regions, with males and individuals over the age of 50 representing the most affected populations (2). Since primary injury is often unavoidable, targeting the secondary injury mechanisms, especially the inflammatory response, is of paramount importance.

Longitudinal studies suggest that cellular debris may persist at the injury site and continuously provoke inflammatory responses, thereby hindering tissue repair. A hallmark pathological feature of SCI is monocyte-derived macrophage (MDM) infiltration and activation of resident microglia within the spinal cord (5). Single-cell mRNA sequencing analyses at various post-injury time points have demonstrated dynamic pathological changes in microglia, astrocytes, endothelial cells, and neurons. Notably, while many cell types tend to revert toward a more primitive or quiescent state during disease progression, microglia appear to undergo lasting, potentially irreversible alterations (6). Macrophages also exhibit phagocytic activity and share considerable morphological and gene expression similarities with activated microglia (5). However, their molecular mechanisms in SCI repair and suitability as treatment targets are not elucidated. Single-cell RNA sequencing (scRNA-seq) enables thorough single-cell characterization at the genomic and transcriptomic levels, thereby enhancing comprehension of cellular heterogeneity, developmental routes, and disease processes (7).

**Figure 1:**
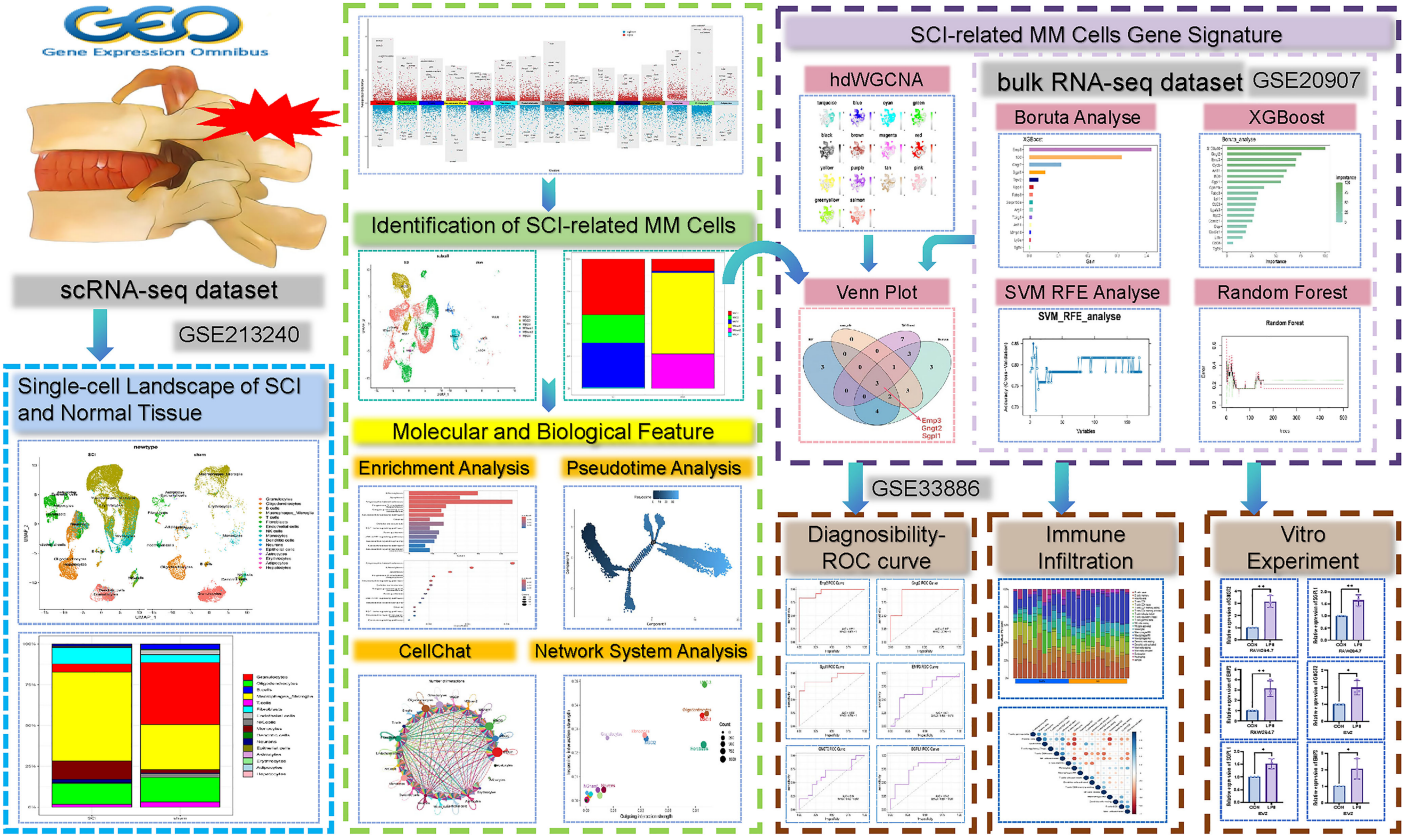
Flow chart: schematic illustration of our analytic approaches.

Previous studies have demonstrated that SCI induces complex alterations in cellular composition and gene expression dynamics (2). Existing literature has highlighted fundamental differences between the two CNS cell types with respect to development, homeostasis, and disease (8). However, the core functions and potential heterogeneity of macrophages and microglia in SCI have not yet been systematically investigated. We hypothesize that, in the setting of SCI, macrophage-microglia (MM) cells differentiate into functionally specialized subpopulations that express distinct gene modules, including characteristic genes that may serve as diagnostic markers for SCI and elucidate key mechanisms underlying post-injury recovery. To validate this hypothesis, our study also involved an in-depth analysis of scRNA-seq data derived from normal and post-injury spinal cord tissues. Our study investigated changes in cellular composition between physi (2)ological and pathological states and examined differentially expressed genes (DEG) across distinct cell populations. Our findings identified six subpopulations of MM cells associated with SCI. High-dimensional weighted gene co-expression network analysis (hdWGCNA) revealed gene modules specific to these subclusters. Subsequently, MM cell-related signature genes implicated in SCI were extracted and validated by four machine learning (ML) algorithms. Receiver operating characteristic (ROC) curve analysis was performed to assess their diagnostic potential, and the correlations between these genes and immune cell infiltration were also explored. *In vitro* experiments were conducted to validate our bioinformatic results(**Figure 1**). Together, our study offers new insights into the biological mechanisms and potential diagnostic biomarkers relevant to post-injury recovery in SCI.

## 2 Materials and methods

### 2.1 Data sources, preprocessing and integration

Our study encompassed the GSE213240, GSE20907, and GSE33886 datasets from the Gene Expression Omnibus (GEO) (https://www.ncbi.nlm.nih.gov/). The GSE213240 dataset contained scRNA-seq information about two normal spinal cord tissue samples and six samples with varying degrees of SCI. The datasets GSE20907 and GSE33886, both related to SCI, were used as the training and external validation sets. GSE20907 includes 22 samples evenly distributed between experimental and control groups, while GSE33886 comprises 14 samples (8 control, 6 experimental), as shown in the **Supplementary File** (**Supplementary Table S1**). Single-cell analysis was enabled by the “Seurat” package (9). Genes expressed in over three cells and cells exhibiting expression of at least 201 genes were further analyzed. Our study excluded cells that showed gene expression below 500 or above 6,000, as well as cells where mitochondrial RNA made up more than a quarter of the content. Dimensionality reduction was performed utilizing the top 4,000 highly variable genes. The initial 20 principal components were obtained via principal component analysis (PCA) through the JackStrawPlot function. Clustering was carried out employing the UMAP algorithm based on these components. To enhance data comparability and improve statistical efficiency, the Harmony package v.1.2.3 (parameters: θ=2, λ=1, dims=1–20) was employed to correct for batch effects. This procedure effectively eliminated technical artifacts and batch-related variability. During this process, a Seurat clustering resolution of 0.4 was applied. Cell sorts were annotated via the “SingleR” package (10) with MouseRNAseqData as a reference. Marker genes were found utilizing the FindAllMarkers function. MM cells were extracted for re-normalization, dimensionality reduction, as well as clustering. Annotation was performed as per their expression across experimental groups.

### 2.2 Enrichment analysis

To unravel the joint effects of genes and their involvement in biological pathways, Gene Ontology (GO) and Kyoto Encyclopedia of Genes and Genomes (KEGG) enrichment analyses were enabled by the “ClusterProfiler” package (11). Moreover, Gene Set Enrichment Analysis (GSEA) demonstrated gene sets possibly displaying no evident differences at the single-gene level but exhibiting strong enrichment trends at the pathway level. This approach offers insights into the overall functional behavior of genes under healthy and diseased conditions (12).

### 2.3 Pseudotime analysis

Pseudotime analysis enables inference of the dynamic progression of cells or samples through a biological process based on gene expression profiles. Monocle, among the most widely adopted tools, constructs developmental trajectories by embedding single-cell data into a low-dimensional space (13). This method facilitated our investigation of the dynamic state transitions of MM cells during SCI progression.

### 2.4 Cell-cell communication analysis

To investigate intercellular signaling, CellChatDB.mouse was the ligand-receptor interaction reference, and the “CellChat” package was applied to infer cell-cell communication networks (14). Interaction frequency and total interaction strength between cell types were visualized using circular plots. The netVisual_bubble function was further employed to display all signaling pathways among cell populations, allowing us to highlight pathways with strong interactions and identify potential signal senders and receivers. This analysis provides insights into intercellular communication, functional cooperation, and responses to external stimuli, offering a deeper understanding of the mechanisms underlying disease development.

### 2.5 hdWGCNA and identification of key genes in MM cells

Unlike conventional scRNA-seq analyses, hdWGCNA facilitates network analysis at the isotype level by leveraging long-read single-cell sequencing information (15). hdWGCNA was executed on MM cells, with the parameter fraction set to 0.05 to retain genes expressed in 5% or more of cells. The TestSoftPowers function presented the ideal soft-thresholding power (Minimum value corresponding to the dimensionless topological fitting index (R^2^≥0.8)). Identifying hub genes - highly interconnected genes within modules - is critical for downstream validation in co-expression network analysis. Therefore, module membership (kME) values were computed using the ModuleConnectivity function and used to identify hub genes within every module. Module-specific hub gene expression levels were measured on a per-cell basis, and modules highly associated with differences between pre-SCI and post-SCI MM cells were selected for further gene extraction.

### 2.6 ML and ROC curve analysis

Candidate disease-associated genes identified via hdWGCNA were further refined using multiple ML algorithms. Four classical algorithms were employed: Random Forest (RF), Survival-SVM, XGBoost, and the Boruta algorithm. These models were integrated to identify shared feature genes. The diagnostic potential of all feature genes and their related nomograms was analyzed through ROC curves with the area under these curves (AUC) as the evaluation standard. An AUC value of approximately 0.65 or higher was considered indicative of moderate to strong predictive performance (16). The GSE142426 dataset was the training set with validation performed on the GSE20907 dataset.

### 2.7 Tumor immune microenvironment

CIBERSORT, an immune cell infiltration analysis tool developed at Stanford University, was employed to analyze the percentages of 22 immune cell sorts among 24 GSE20907 samples (17). Subsequently, Pearson correlation coefficients revealed the relation of characteristic gene expression to immune cell subset infiltration.

### 2.8 *In vitro* experiments

RAW264.7 macrophages and BV-2 microglial cells were cultured in standard conditions (37°C, 5% CO_2_). The control group (CON group) consisted of cells grown for 48 hours in high-glucose DMEM comprising 10% fetal bovine serum and 1% penicillin/streptomycin. In contrast, those treated for 48 hours with a medium containing 1 mg/mL lipopolysaccharide (LPS) served as the inflammation-mimicking model group (LPS group). Gene expression levels of GNGT2, EMP3, and SGPL1 in both groups were quantified via real-time qPCR (18). The Trizol reagent was employed for the isolation of total RNA, which was then converted into complementary DNA (cDNA) via the Seven All-in-one First Strand cDNA Synthesis Kit II (SM134). Amplification was enabled by the Seven SYBR Green qPCR MasterMix II (SM143). mRNA was quantified with β-Actin as the reference. Relative gene expression was calculated through the 2^−ΔΔCt approach. Statistical significance was evaluated via independent sample t-tests, with P < 0.05 denoting statistical significance.

The primer sequences were: CD206: forward 5’-GTTCACCTGGAGTGATGGTTCTC-3’, reverse 5’-AGGACATGCCAGGGTCACCTTT-3’ IL-10: forward 5’-CGGGAAGACAATAACTGCACCC-3’, reverse 5’-CGGTTAGCAGTATGTTGTCCAGC-3’ TGF-β: forward 5’-TGATACGCCTGAGTGGCTGTCT-3’, reverse 5’-CACAAGAGCAGTGAGCGCTGAA-3’ β-Actin: forward 5’-ACTGCCGCATCCTCTTCCT-3’, reverse 5’-TCAACGTCACACTTCATGATGGA-3’ GNGT2: forward 5’-GCTGTTGAGGATGGAGGTG-3’, reverse 5’-AAGGGATTCTTGTCTTCTGG-3’ SGPL1: forward 5’-GGAAAGCCTCAGGAGCTGTGTA-3’, reverse 5’-CTGCCTCTAACTTCCGCAATCC-3’ EMP3: forward 5’-GTTCCAACTCTACACCATGCGG-3’, reverse 5’-ATCTCCTCGGTGTGGATGGCAT-3’.

### 2.9 Statistical analysis

Our data analysis was enabled by R 4.4.1. Experimental data were statistically studied via GraphPad Prism (GraphPad Software, La Jolla, CA). Inter-group difference assessment was completed through independent sample t-tests. Asterisks denote the following: *** P < 0.001, ** P < 0.01, * P < 0.05.

## 3 Results

### 3.1 Single-cell transcriptomic profiles of MM cells in normal versus SCI tissue

**Figure 2:**
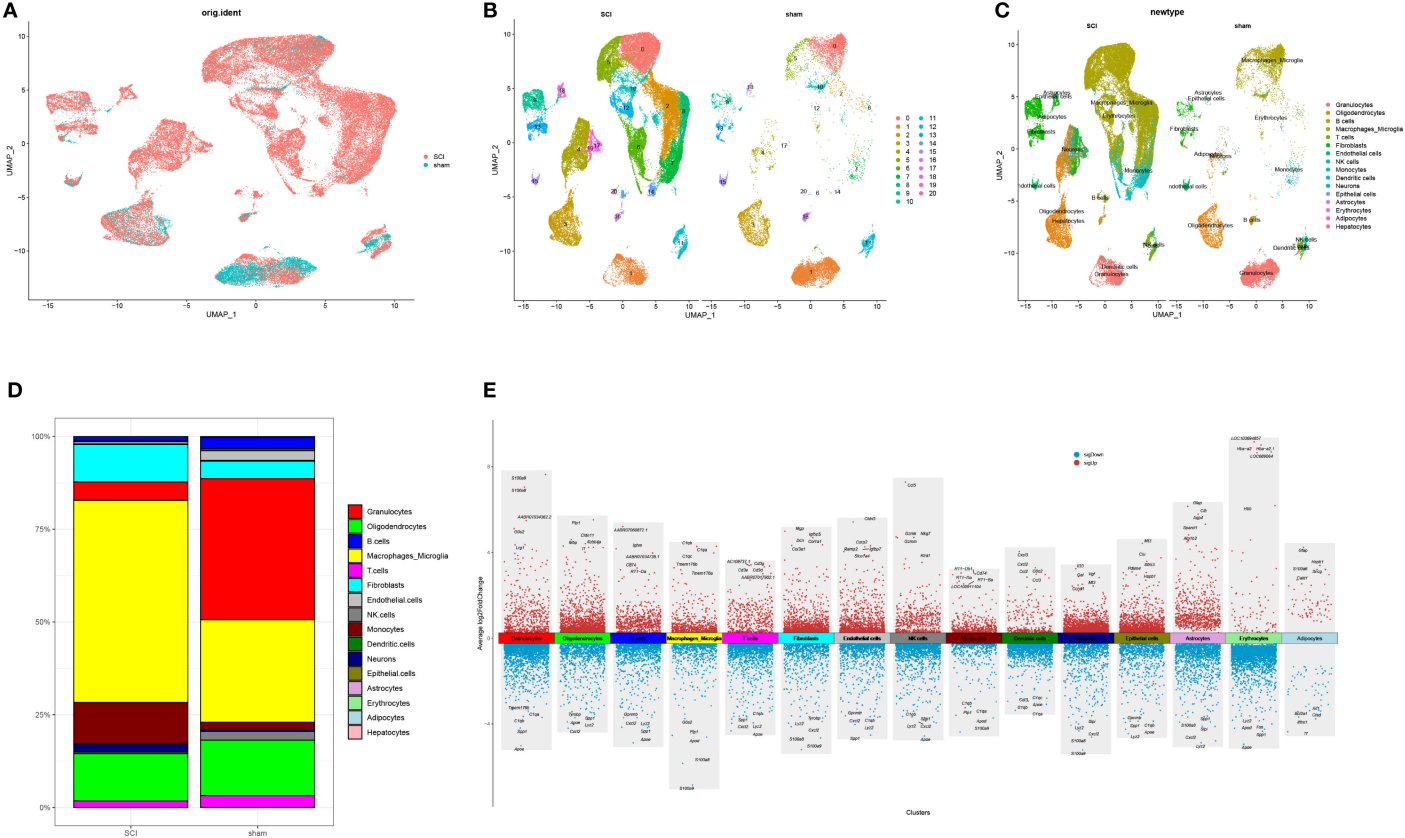
Cellular composition analysis of tissues in SCI **(A)** UMAP visualization depicting the distribution of cells in normal tissues and SCI samples. **(B)** Identification of distinct cell clusters. **(C)** Classification of major cell types. **(D)** Proportions of different cell types in normal and SCI tissues. **(E)** Marker genes of 15 identified cell clusters.

Following quality checks and batch effect correction, 58,674 high-quality cells expressing 19,414 genes were retained. Among these, the SCI group contributed 46,650 cells expressing 18,874 genes, while the normal group provided 12,024 cells expressing 18,158 genes. Unsupervised clustering identified 21 distinct cell clusters (**Figures 2A, B**). Subsequent annotation revealed 16 well-defined cells like granulocytes, oligodendrocytes, macrophages/microglia, fibroblasts, monocytes, B, T, endothelial, NK, dendritic and epithelial cells, neurons, astrocytes, erythrocytes, adipocytes, and hepatocytes (**Figure 2C**). The proportions of each cell type were compared across the control and experimental cohorts (**Figure 2D**). The percentage of MM cells was notably increased following SCI. Differential gene expression analysis was subsequently performed with results visualized via volcano plots (**Figure 2E**, **Supplementary Table S2**).

**Figure 3:**
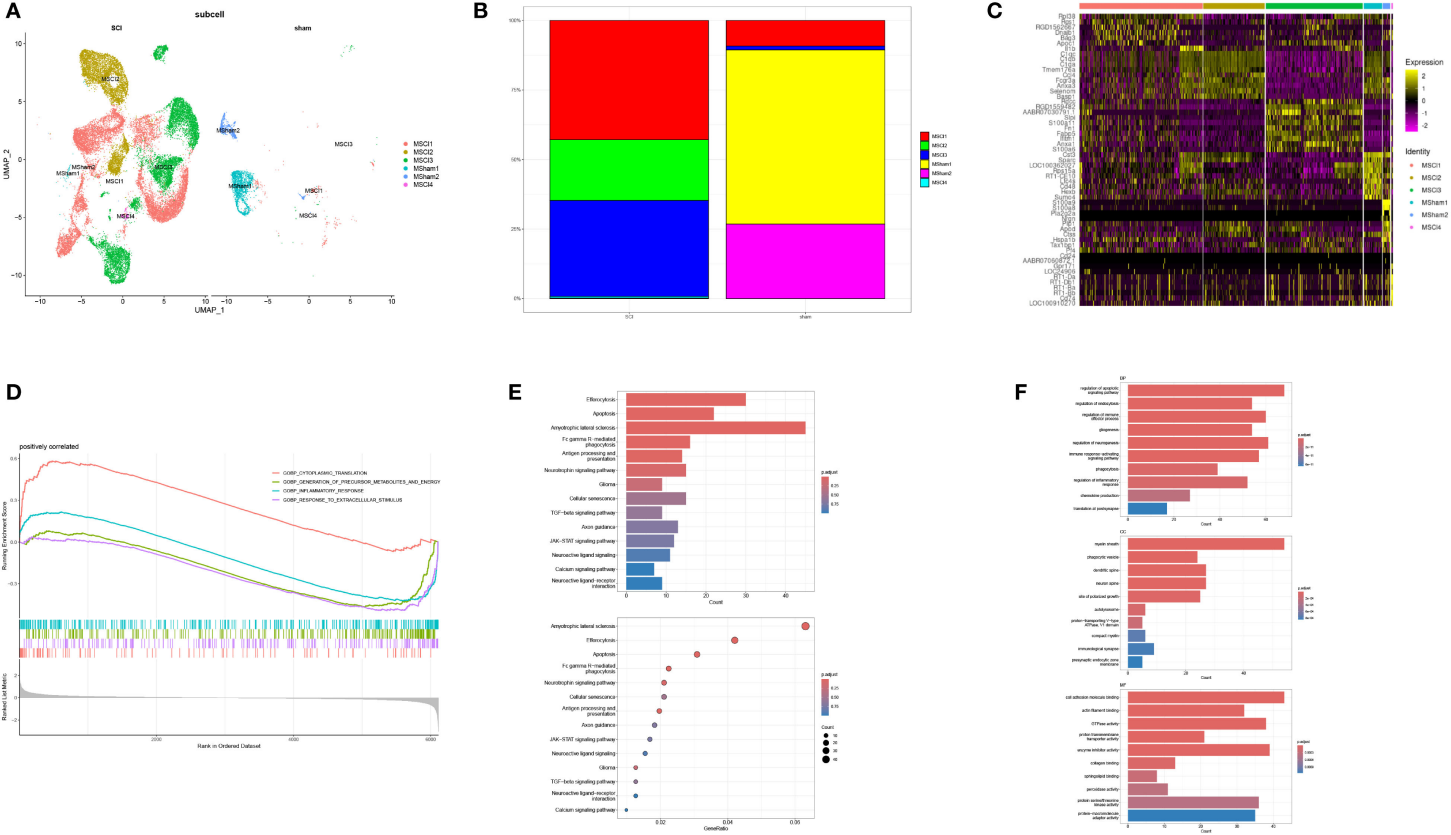
Subtype identification of MM cells and functional enrichment analysis of associated genes. **(A)** UMAP visualization depicting the clustering of MM cells in both normal and diseased samples. **(B)** Proportional distribution of MM cell subpopulations in normal tissue and SCI samples. **(C)** Expression profiles of subtype-specific marker genes across MM cell subgroups. **(D)** GSEA of the MSCI1 and MSCI3 subpopulations. **(E, F)** GO and KEGG enrichment analyses, respectively.

An in-depth MM cell analysis was further carried out. Following repeated UMAP analysis, six cellular subpopulations were identified via manual annotation, with the classification being determined by the differential gene expression observed across the two tissue varieties (**Figure 3A**). Four of the six subpopulations (MSCI1, MSCI2, MSCI3, and MSCI4) exhibited elevated expression levels following SCI. Therefore, subsequent investigations were directed toward these four subpopulations. Subsequent statistical analysis revealed significant differences in the MSCI1 and MSCI3 subclusters (**Figure 3B**), suggesting the crucial roles of these two cell populations in SCI onset and progression. A heatmap further presented the expression patterns of subtype-specific marker genes within MM cells (**Figure 3C**). To unravel the possible molecular mechanisms involving MSCI1 and MSCI3 in SCI, GSEA was performed on significantly altered gene sets. The results indicated substantial activation of pathways related to cytoplasmic translation, inflammatory response, precursor metabolite, energy production, and response to external stimuli (**Figure 3D**). Additionally, GO and KEGG enrichment analyses revealed significant involvement in biological processes such as regulation of the nerve growth factor pathway, adaptive immune response, phagocytic apoptosis signaling, inflammatory response, and in the pathogenesis of diseases, including glioma (**Figures 3E, F**).

### 3.2 Pseudotime trajectory and cell-cell interaction analysis

**Figure 4:**
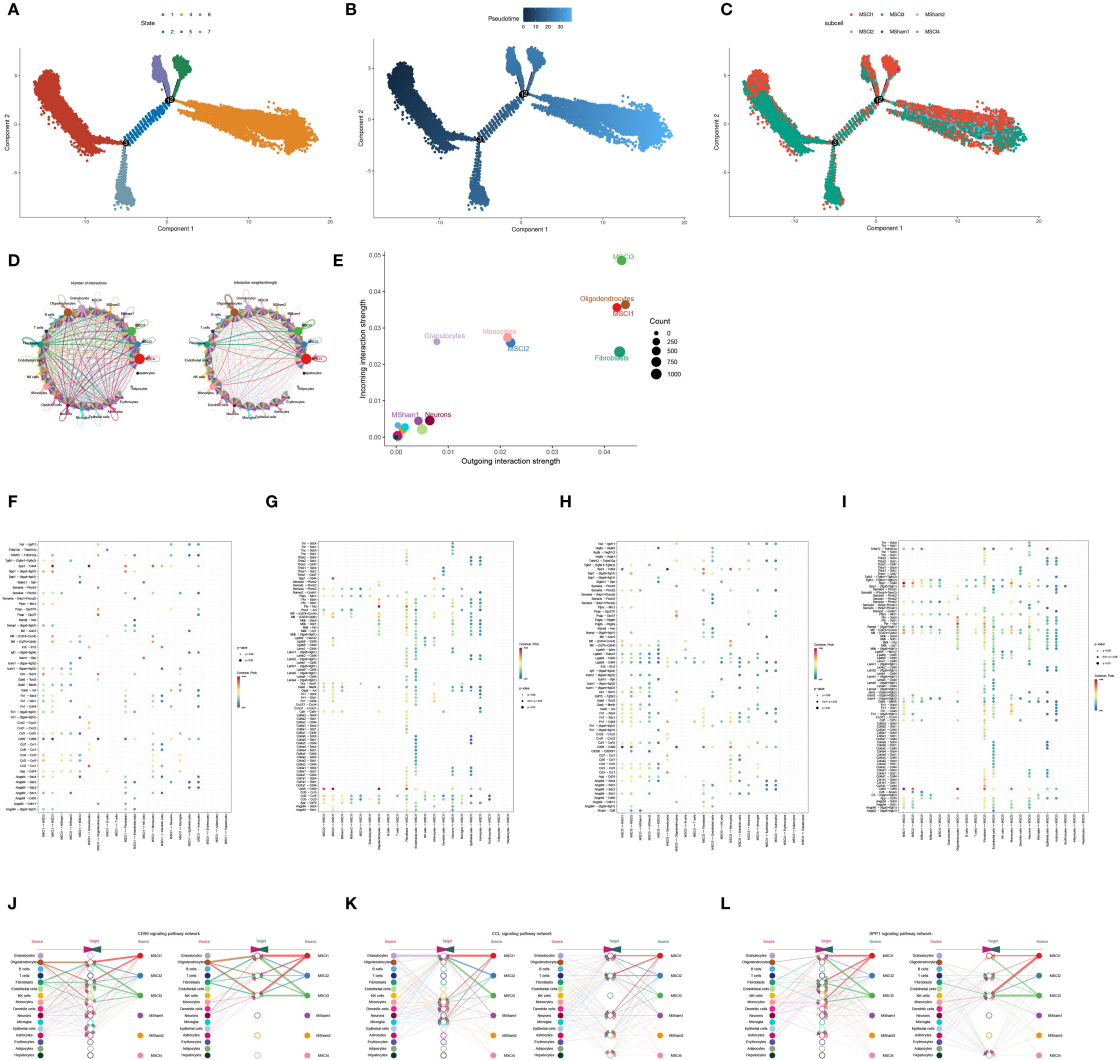
Trajectory and intercellular communication analysis of MM cell clusters in SCI. **(A–C)** The dynamic trajectory of macrophages during SCI progression is illustrated from three perspectives: cellular state, pseudotime, and subcellular localization. **(D)** The number and intensity of ligand-receptor interactions demonstrate significant intercellular communication across all identified cell populations. **(E)** Sender and receiver signal intensities are visualized across all cell types in a two-dimensional space. **(F–I)** The average expression levels and statistical significance of cytokine-related signaling interactions between MSCI1, MSCI3, and other cell populations are depicted. **(J–L)** Hierarchical plots illustrate the cellular communication networks characterized by high expression of CD99, CCL, and SPP1 signaling pathways.

Pseudotime trajectory analysis revealed the dynamic changes in MM cells during the advancement of SCI. This analysis clearly illustrated that, with disease progression, the developmental trajectory of these cells can be delineated into seven distinct phases (**Figure 4A**). The inferred pseudotemporal trajectory was constructed based on integrated functional metrics (**Figure 4B**). Upon annotation of the cell subpopulations, it became evident that the MSCI1 and MSCI3 subsets play prominent roles throughout disease progression (**Figure 4C**).

Given these findings, MSCI1 and MSCI3 were selected for further in-depth investigation. To systematically explore the interactions between MSCI1, MSCI3, and other MM cell subpopulations, our study employed the CellChat algorithm, which integrates the expression levels of signaling ligands, receptors, and their cofactors to infer intercellular communication networks. The analysis revealed robust and extensive interactions between MSCI1, MSCI3, and other cellular subtypes (**Figure 4D**). Notably, MSCI1 and MSCI3 exhibited enhanced secretory activity and demonstrated greater outgoing interaction strengths compared to other subgroups (**Figure 4E**). The investigation of ligand-receptor pairing between MSCI1/MSCI3 and different cells uncovered several highly expressed molecular axes, including CD99-CD99, SPP1-CD44, and CCL3-CCR1 (**Figures 4F–I**). Further analysis indicated that signaling pathways associated with immune response, inflammatory processes, and cell migration, particularly those involving CD99, CCL3, and SPP1, were markedly upregulated in communications involving MSCI1 and MSCI3 (**Figures 4J–L**).

### 3.3 hdWGCNA and module-specific hub gene identification

**Figure 5:**
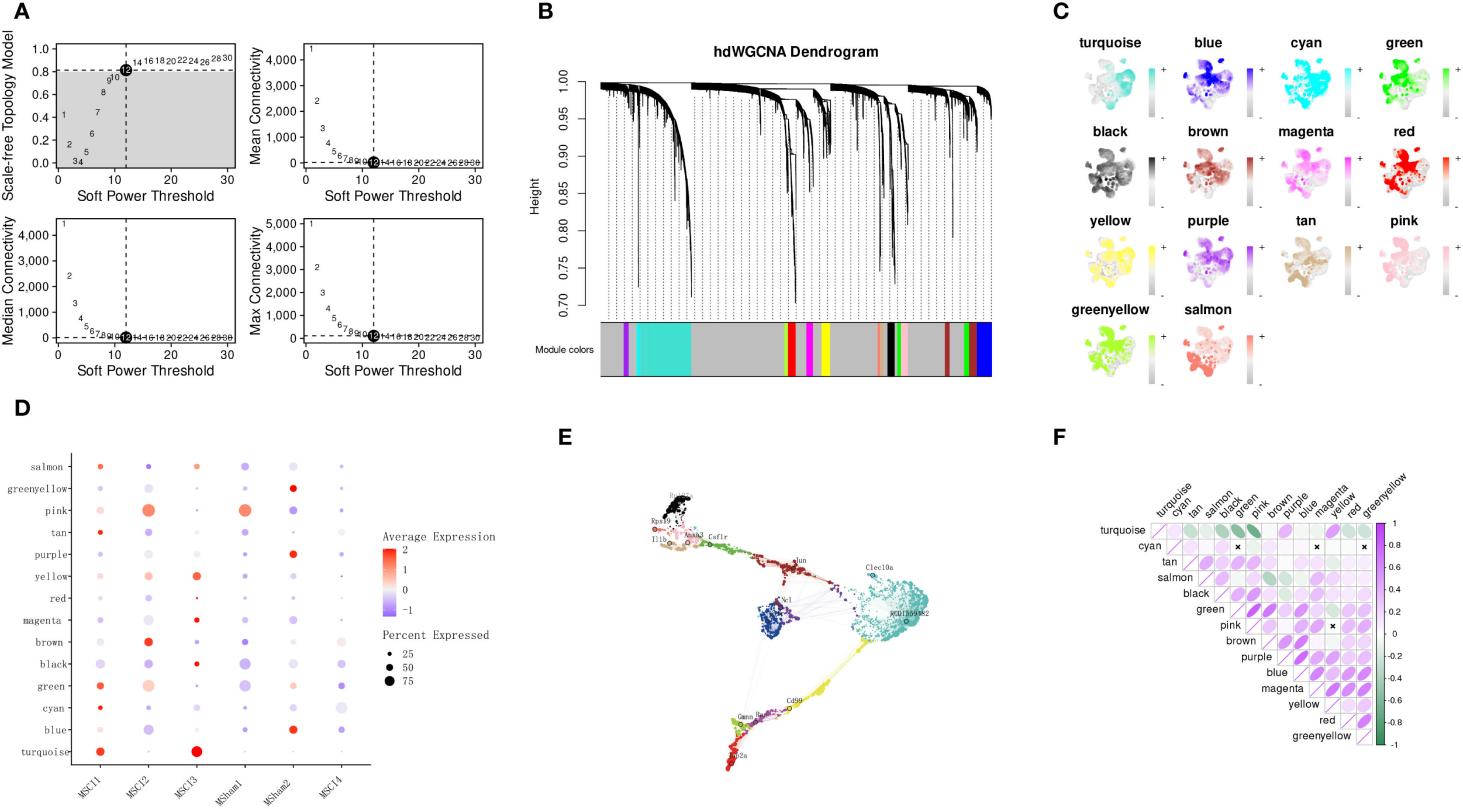
Identification of hub modules associated with SCI-related MM cells using hdWGCNA. **(A)** Selection of the optimal soft-thresholding power. **(B)** Construction of the co-expression network and visualization of the hierarchical clustering dendrogram with gene modules (hMEs). **(C)** Visualization of module eigengene values for each cell based on the UMAP projection of MM cell subpopulations derived from scRNA-seq analysis. **(D)** Expression patterns of different hMEs across distinct phagocytic cell populations. **(E)** Visualization of the co-expression network among gene modules, with annotation of the top hub gene in each module. **(F)** Heatmap depicting the correlations between different gene modules.

To ensure biological relevance in the WGCNA, a soft-thresholding power of 12 was used to approximate a scale-free topology (**Figure 5A**). Upon achieving optimal scale - Independence and mean connectivity, 14 gene modules were noted (**Figure 5B**). Within each module, highly connected genes - termed hub genes - were determined by calculating the link of individual genes to the module eigengene (KME value). Notably, the turquoise module was considerably enriched in MSCI1 and MSCI3 (**Figure 5C**). The expression activity of hub genes within each module was visualized across individual cells (**Figure 5D**). Furthermore, our study examined the inter-subgroup expression patterns of each module (**Figure 5E**) and the inter-module correlation structure (**Figure 5F**). The turquoise module exhibited a positive correlation with the purple and yellow modules, and prior cell interaction analysis demonstrated that MSCI1 and MSCI3 were strongly associated with oligodendrocytes and fibroblasts. **Figures 4E, F** indicate that Ptn-Ncl and Cd99-Cd99 are highly expressed. The expression of the Ptn and Cd99 genes was subsequently validated (**Supplementary Figure S1**), thereby corroborating this observation and providing insight into potential novel signaling pathways between these cell types.

### 3.4 ML-based identification of diagnostic markers in SCI-associated macrophages and evaluation of their diagnostic potential

**Figure 6:**
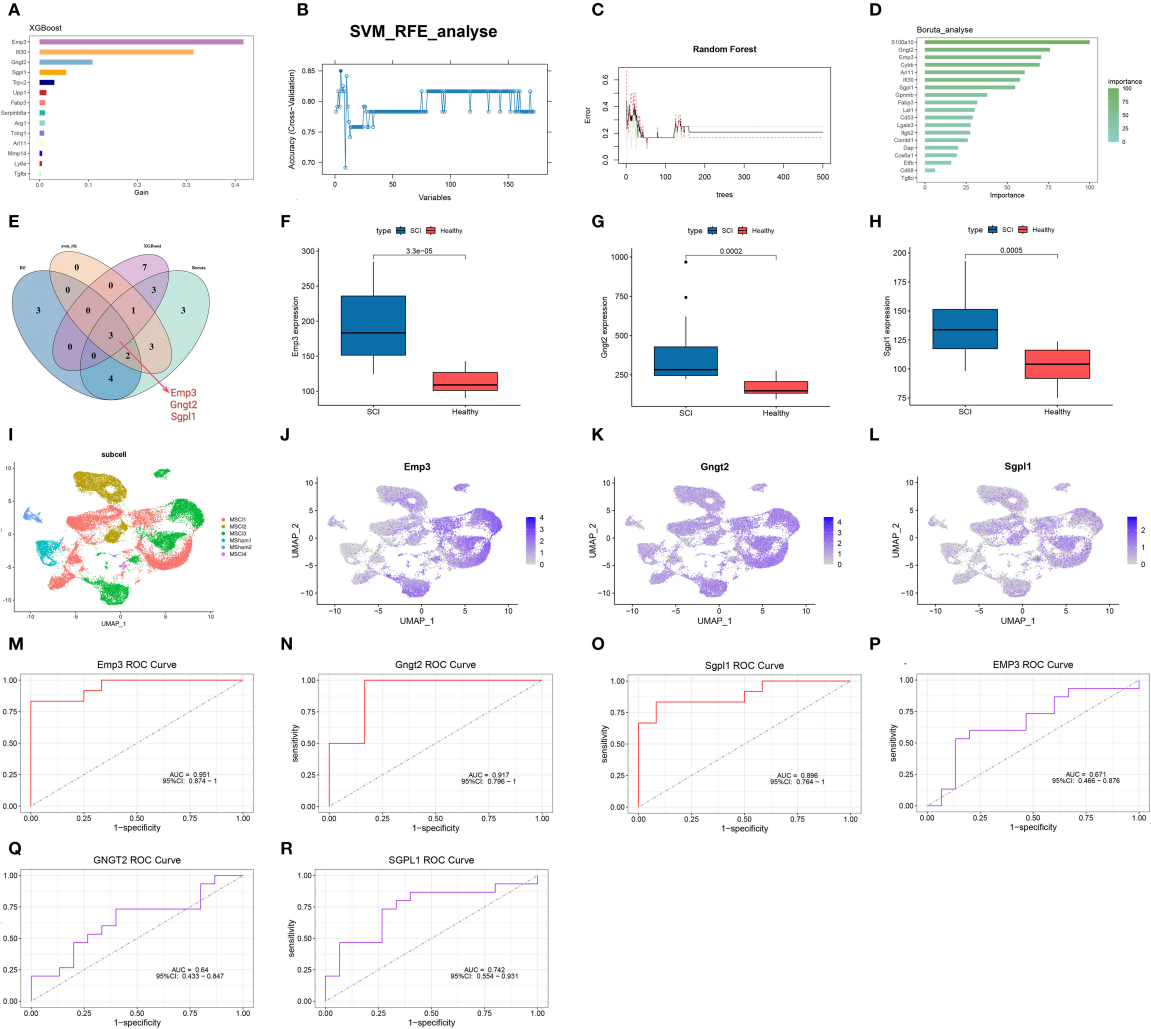
Identification of key genes via ML algorithms. **(A)** Gene ranking based on feature importance in the XGBoost model. **(B)** Accuracy curve from cross-validation using the SVM-RFE algorithm. **(C)** Error curve from random forest analysis. **(D)** Key gene selection using Boruta analysis. **(E)** Venn diagram displaying three overlapping genes obtained from intersecting results. **(F–H)** Boxplots depicting differential expression of the three genes. **(I–L)** Gene expression patterns visualized via UMAP. **(M–R)** ROC curves illustrating the predictive performance of EMP3, GNGT2, and SGPL1 in SCI.

In the previous analysis, 200 hub genes were identified within the turquoise module. Building upon these results, four ML algorithms were further employed to mine for SCI-associated central genes using the GSE20907 dataset. In the XGBoost algorithm, parameters were set as max_depth=5 and eta=0.2, and the top 14 genes were identified based on the feature importance evaluation (**Figure 6A**). Subsequent SVM-RFE analysis indicated that prediction efficacy was greatest with nine genes (**Figure 6B**). The RF model displayed the lowest misclassification rate when the number of decision trees reached 53, identifying 12 candidate genes at this point (**Figure 6C**). Using parameters (perc = 100, alpha = 0.01, max_features < 0.3) in Boruta analysis, 19 genes that significantly contributed to the prediction task were effectively screened out (**Figure 6D**). Venn diagrams present the overlapping genes identified through the four algorithms, resulting in the selection of three intersecting genes: EMP3, GNGT2, and SGPL1 (**Figure 6E**).

After comparing spinal cord tissue datasets before and after injury, EMP3, GNGT2, and SGPL1 were markedly upregulated following SCI (**Figures 6F–H**). Integration of spatial transcriptomic data with single-cell gene expression patterns demonstrated the main enrichment of these three genes in the MSCI1 and MSCI3 clusters (**Figures 6I–L**) (**Supplementary Figure S2**). To evaluate their diagnostic potential, ROC curve analyses were carried out for EMP3, GNGT2, and SGPL1, which yielded AUC values of 0.951, 0.917, and 0.896 (**Figures 6M–O**). Validation using an external dataset (GSE33886) demonstrated AUCs of 0.671, 0.640, and 0.742 (**Figures 6P–R**).

### 3.5 Immune infiltration analysis

**Figure 7:**
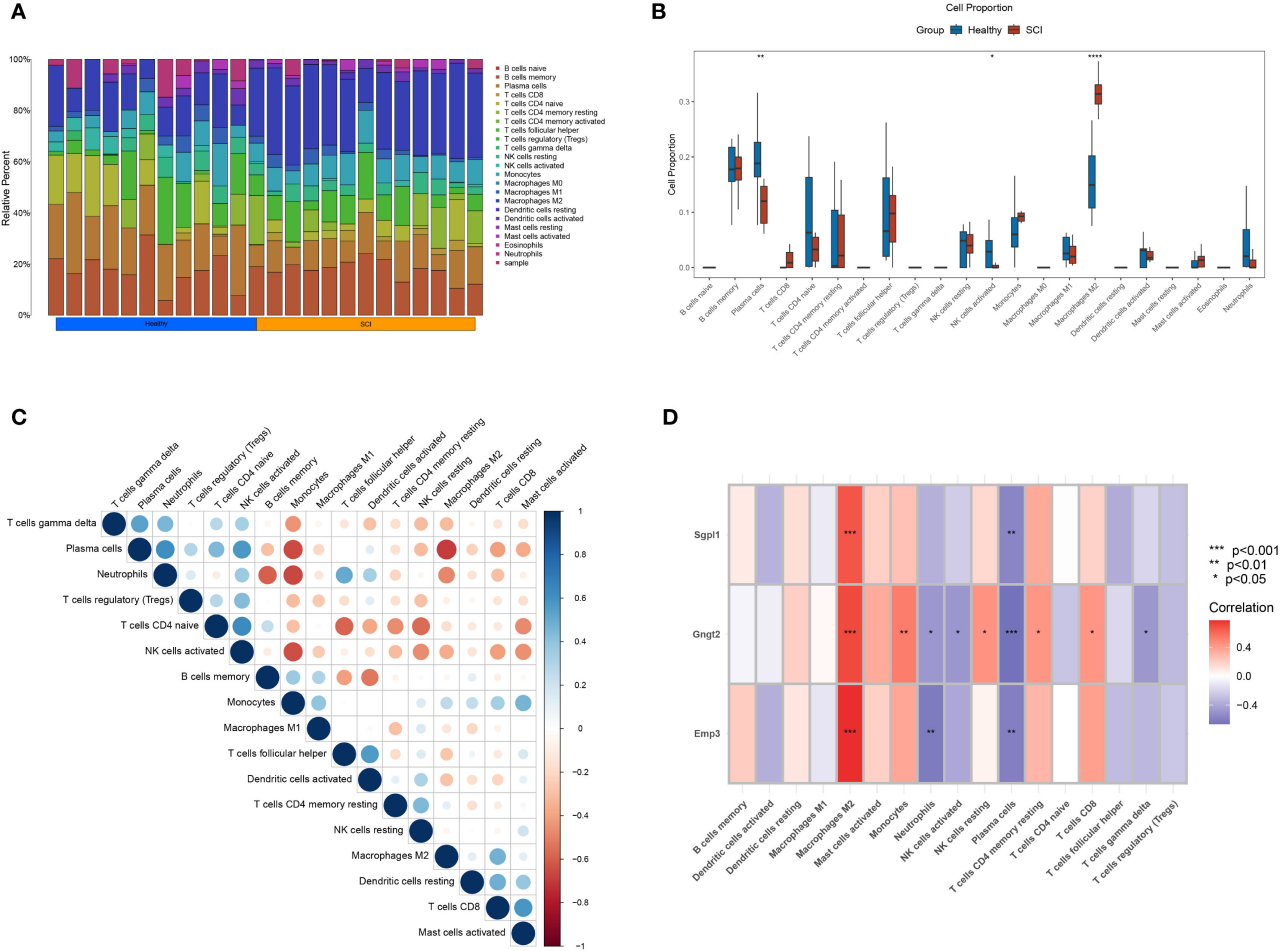
Immune infiltration landscape. **(A)** Proportional differences of 22 immune cell types across individual samples. **(B)** Box plots illustrating the differential abundance of immune cells. **(C)** Correlation matrix depicting the relationships among immune cell populations. **(D)** Correlation coefficients and P-values between SGPL1, GNGT2, EMP3, and various immune cell types.

Infiltration of immune cells, which is crucial for inflammatory reactions, significantly and independently influences the pathophysiology of SCI. CIBERSORT, a computational method for estimating the relative abundance of immune cell sorts within a single sample, was employed via the LM22 signature matrix, which profiles 22 immune cell subsets with diverse phenotypes and functional states. Comparative analysis revealed marked differences in macrophage infiltration between normal and SCI tissues, suggesting pronounced macrophage involvement in SCI samples (**Figures 7A, B**). The intercorrelations among all immune cell types were further analyzed (**Figure 7C**). M2 macrophages were found to be positively correlated with CD8 T cells, while exhibiting a negative correlation with plasma cells, neutrophils, and activated NK cells. In addition, activated NK cells, plasma cells, and naïve CD4 T cells demonstrated positive correlations. These observations provide preliminary insights into the interactions among immune cell subsets. Using expression data of the genes identified via ML and the immune cell infiltration matrix, our study examined the associations between SGPL1, GNGT2, EMP3, and immune cells. Notably, all three genes exhibited strong correlations with M2 macrophages (**Figure 7D**), providing theoretical support for targeting M2 macrophages in future studies on therapeutic strategies for SCI recovery.

### 3.6 *In vitro* models

**Figure 8:**
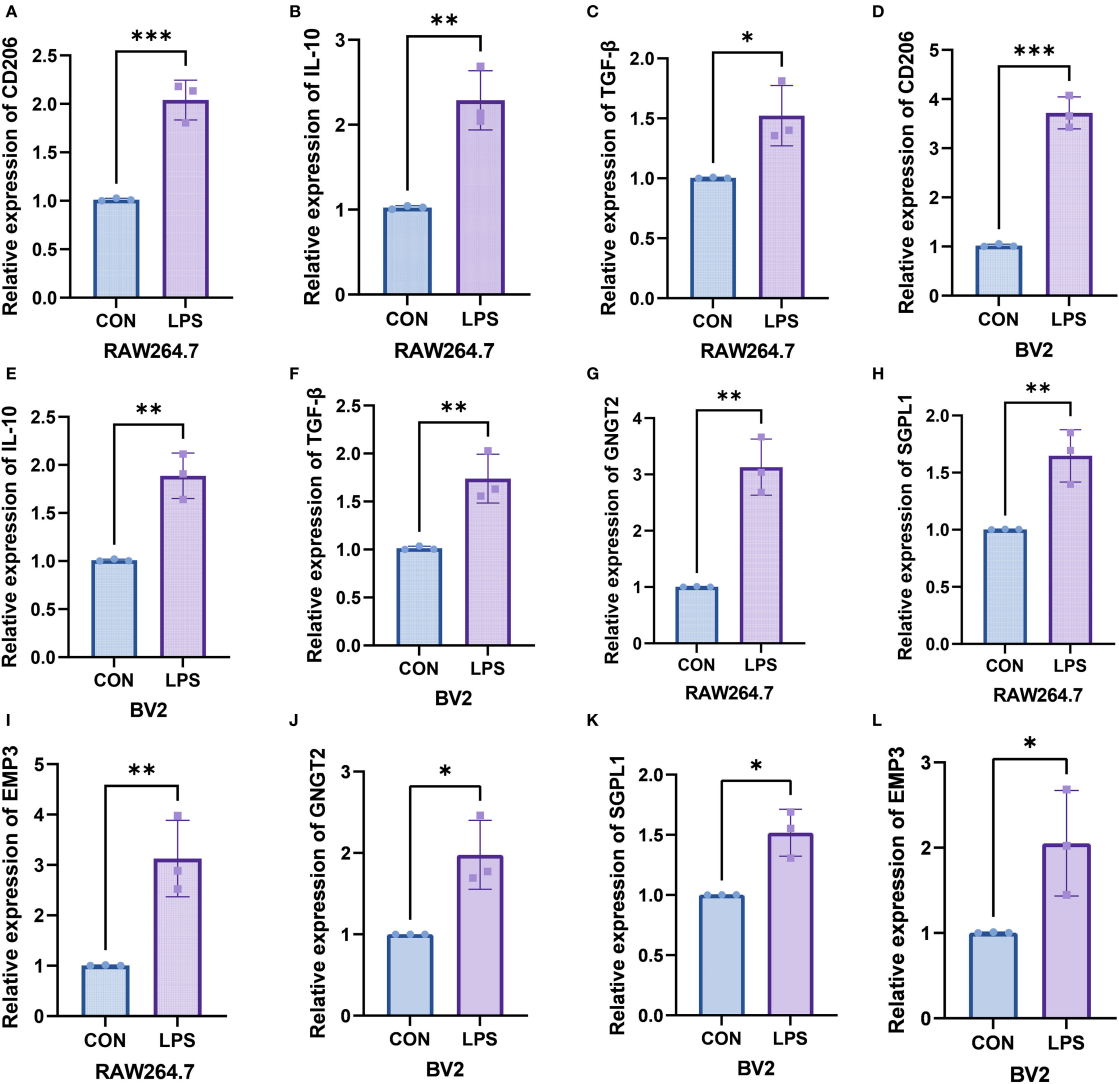
*In vitro* experiments validated the expression of the target genes. **(A–C)** Relative mRNA levels of CD206, IL-10and TGF-β in RAW264.7 macrophages. **(D–F)** Relative mRNA levels of CD206, IL-10, and TGF-β in BV-2 microglial cells. **(G–I)** Relative mRNA levels of GNGT2, SGPL1, and EMP3 in RAW264.7 macrophages. **(J–L)** Relative mRNA levels of GNGT2, SGPL1, and EMP3 in BV-2 microglial cells. ***P < 0.001, **P < 0.01, *P < 0.05.

In the RAW264.7 macrophage *in vitro* inflammation model, the mRNA levels of CD206 (P = 0.001), IL-10 (P = 0.0033), TGF-β (P = 0.0233), GNGT2 (P = 0.0018), SGPL1 (P = 0.0082), and EMP3 (P = 0.0083) were markedly upregulated in the LPS-treated group. Similarly, in the BV-2 microglial inflammation model, elevated expression trends were also observed for CD206 (P = 0.0001), IL-10 (P = 0.003), TGF-β (P = 0.0078), GNGT2 (P = 0.0163), SGPL1 (P = 0.0101), and EMP3 (P = 0.0426). The corresponding qPCR results are presented in **Supplementary Tables S3–S6**, and **Figure 8**.

## 4 Discussion

The spinal cord, part of the CNS connecting the brain to various body parts, is vital in communication and coordination. Its injury leads to a series of severe consequences. With ongoing exploration into disease mechanisms, it has become evident that MM cells play an irreplaceable role in subsequent inflammatory responses and immune regulation (5, 19). However, few studies unveiled the differential expression of gene biomarkers in cells before and after SCI. The present study integrates single-cell data with large-scale bulk RNA sequencing to provide a more comprehensive comparison of SCI and normal samples. Enrichment and cell-cell communication analyses identified potential biological mechanisms and novel signaling pathways. Further investigation through hdWGCNA and multiple machine learning algorithms indicated that SGPL1, EMP3, and GNGT2 may play pivotal roles in SCI, thereby offering new perspectives on the molecular underpinnings of SCI.

In the samples studied, MM cells comprised nearly half of the population following SCI, consistent with previous findings (19). Various enrichment analyses of these cells indicated their association with immune responses, inflammation, apoptosis, and other biological behaviors. Further investigation identified that the activated MM cells exhibit abnormal regulation of several signaling pathways, such as SPP1, CCL3, and CD99. Notably, SPP1 (osteopontin; OPN) secreted by immune cells, is crucial in pathological vascular remodeling post-SCI, with studies demonstrating its activation of the Itgav/PI3K/Akt signaling pathway for early vascular regeneration (20). CD11c+ microglia are the main source of OPN, and OPN, through binding to its classic receptor αVβ3 integrin, facilitates phagocytosis and inflammatory responses (21). Research by Sebastiaan De Schepper and colleagues in an Alzheimer’s disease model suggests that extracellular SPP1 may also regulate synaptic phagocytosis by microglia via TGF-β signaling (22). Within the CNS, microenvironmental inflammatory signals polarize macrophages toward pro-inflammatory (M1) or anti-inflammatory (M2) types (23). M1 aggravates cellular damage and hinders tissue repair by releasing destructive inflammatory cytokines, whereas M2 facilitates tissue repair and mitigates inflammatory reactions by clearing necrotic debris and releasing protective factors (24, 25). However, recent studies have demonstrated that microglia remain active even in normal brain tissue, prompting scholars to question the traditional M1/M2 classification. A more precise and nuanced terminology is therefore warranted. Since inflammation cannot invariably be regarded as detrimental, the term “homeostatic” should be applied cautiously with an accurate description of the SCI context (26–28). CCL3, a pro-inflammatory chemokine linked to macrophages, can trigger macrophage polarization toward M1 via upregulation of the Ccr5-p38/Irf5 pathway (29), and stimulate the generation of other pro-inflammatory cytokines via receptors coupled to G protein: CCR 1, 4, and 5 (30). Notably, CCL3 deficiency has been shown to improve functional recovery, lesion size, and the intensity of inflammation following SCI (31). In this study, CCL3-CCR1 signaling is highly regulated after SCI, though the exact mechanisms require further exploration. In studies on Ewing Sarcoma, the activation of CD99 in macrophages was shown to induce the secretion of characteristic chemokines such as IL1β, IL6, TNFα, and markers CD80 and CD86 by M1 macrophages (32). Therefore, modulation of macrophage phagocytic capacity and promotion of M2 polarization represent important future directions for this study. Our future investigations should address not only cell polarization but also the homeostatic balance between the two cell types.

Moreover, after employing hdWGCNA and screening with four ML algorithms (XGBoost, SVM-RFE, RF, and Boruta), three key genes, namely SGPL1, EMP3, and GNGT2, were identified. Validation in both training and test sets revealed an AUC range of 0.64 to 0.951, indicating their reliability and relevance. SGPL1, an enzyme involved in the last step of sphingolipid catabolism, degrades its substrate S1P irreversibly. Astrocytes with SGPL1 deficiency release accumulated S1P, which can indirectly activate microglia and boost the generation of pro-inflammatory cytokines (33). Rapamycin has been shown to partially suppress inflammatory responses in microglia (34), raising the possibility that rapamycin may have potential therapeutic effects in SCI patients during recovery. Furthermore, released S1P can activate volume-regulated anion channels (VRAC), which are ATP release channels, linking microglia activation and neuropathic pain to extracellular ATP-mediated purinergic signaling (35). EMP3, a transmembrane protein widely expressed in various cells, particularly epithelial cells, has gained attention for its role in immune cells, especially macrophages. In previous research on glioblastoma, Qun Chen and colleagues found that EMP3 promotes the migration and M2 polarization of monocyte-derived macrophages and downregulates the production of chemokines induced by interferon, CXCL9 and CXCL10, by macrophages (36). Interestingly, CXCR3, a binding site for CXCL9 and CXCL10, is expressed on the surface of monocyte-derived macrophages (37). Inhibition of EMP3 expression may reduce its intrinsic positive feedback regulation, thereby minimizing macrophage recruitment in the initial phase of SCI and the inflammatory damage. Although research on GNGT2 in the nervous system remains limited, its role in biological regulation cannot be overlooked. However, the effects of upregulation or downregulation of these three genes in SCI on macrophage regulation and prognosis warrant further investigation.

The primary immune cells exhibiting significant differential infiltration in the samples were M2 macrophages and plasma cells. Furthermore, an evaluation of the correlation among 22 immune cell sorts demonstrated a positive relation of activated NK and plasma to naive CD4+ T cells, but a negative correlation was observed between M2 macrophages and plasma cells. These findings, in conjunction with the study of key genes and their link to immune cells, prove the hypothesis that the levels of these three critical genes may influence the immunological activity of immune cells. Furthermore, by culturing two types of cells *in vitro* and simulating the inflammatory environment following SCI, we observed a significant upregulation of the three genes after injury. This finding not only corroborates our previous results but also provides a solid theoretical basis for our subsequent in-depth mechanistic investigations.

This study further explores, from a molecular perspective, the signaling pathways and genetic states associated with SCI concerning MM cells. ML algorithms suggest that SGPL1, EMP3, and GNGT2 may be key molecules involved in progression, with evidence of their correlation with immune cells, particularly M2 macrophages. However, our study has limitations. Given the limited scRNA-seq data and the unclear precise regulation mechanisms of the three genes, these aspects will require further investigation and refinement in subsequent studies.

## 5 Conclusions

In summary, this study comprehensively describes three key genes (SGPL1, EMP3, and GNGT2) associated with MM cells after SCI and evaluates their diagnostic performance (AUC 0.64-0.951). The findings also provide preliminary evidence for their association with M2 macrophages, opening new avenues for fundamental research and potential therapeutic strategies in SCI.

## Data Availability

Publicly available datasets were analyzed in this study. This data can be found here: Our study encompassed the GSE213240, GSE20907, and GSE33886 datasets from the Gene Expression Omnibus (GEO) (https://www.ncbi.nlm.nih.gov/).

## Supplementary material

Supplementary Figure 1 Expression of Ptn and Cd99 in 16 cell types.

Supplementary Figure 2 The expression levels of the selected genes (Emp3, Gngt2, and Sgpl1) in six subpopulations of MM cells.

Supplementary Table 1 Detailed information on the datasets.

Supplementary Table 2 Marker genes in 15 cell types.

Supplementary Table 3-6 qPCR analysis.
