# Supplementary figures and images for: Novel diagnostic biomarkers associated with macrophage-microglia in spinal cord injury
Source: Front Immunol. 2025 Sep 10;16:1634014. doi: 10.3389/fimmu.2025.1634014 (PMC12457113; doi:10.3389/fimmu.2025.1634014)

Ptn

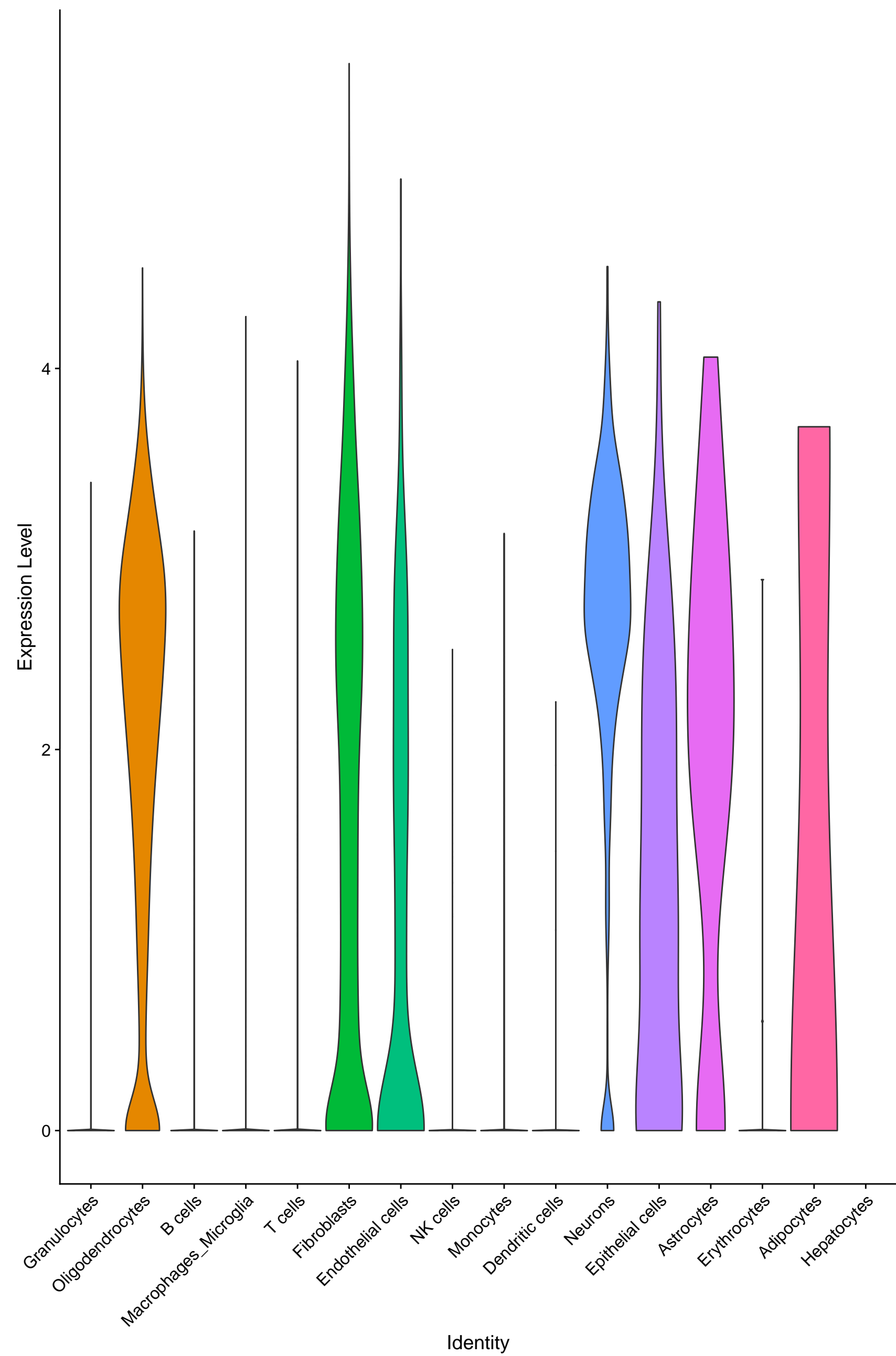

Cd99

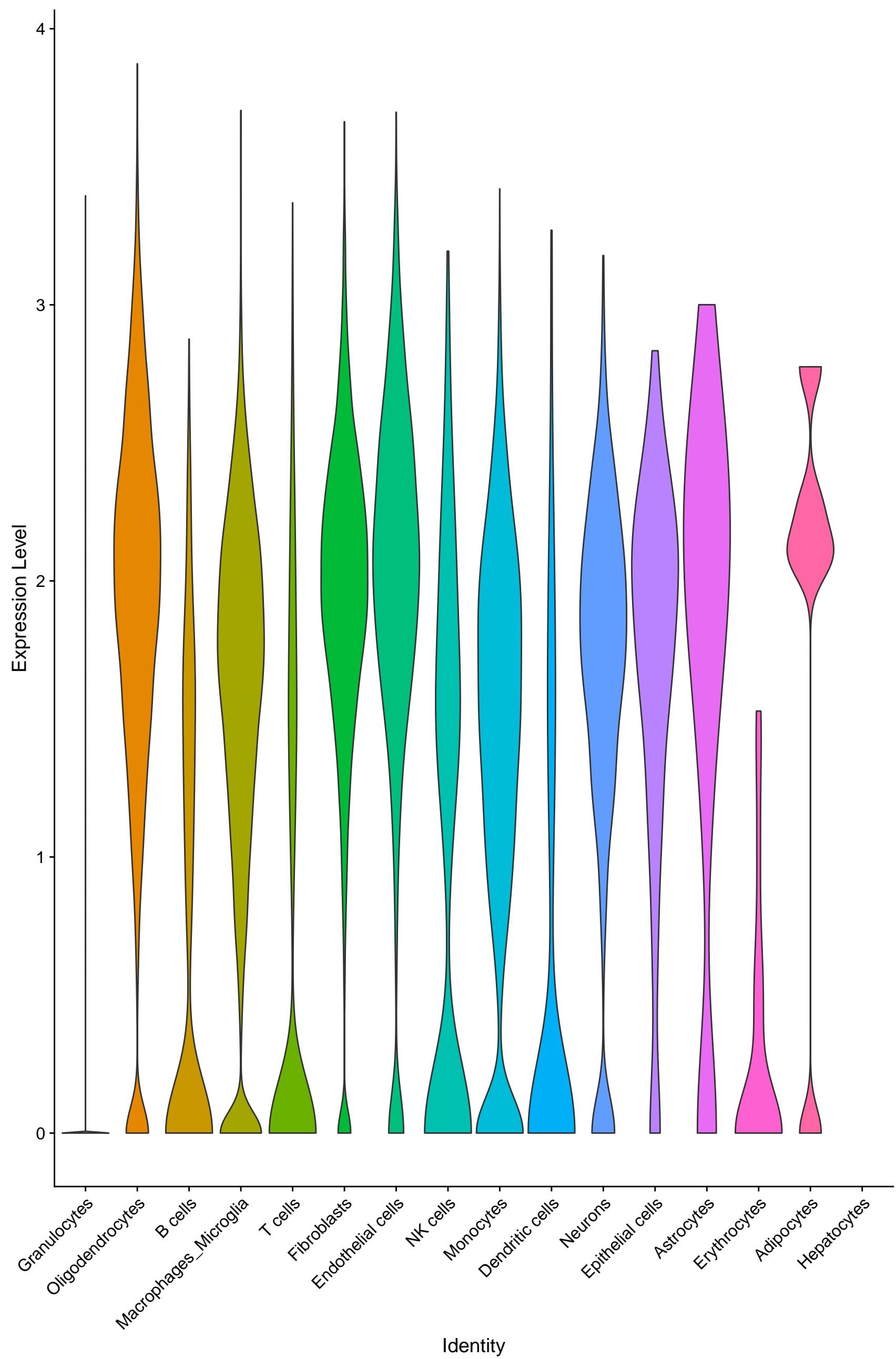

Supplement: Supplementary Figure 1 — Expression of Ptn and Cd99 in 16 cell types. [file DataSheet1.pdf]

**Emp3**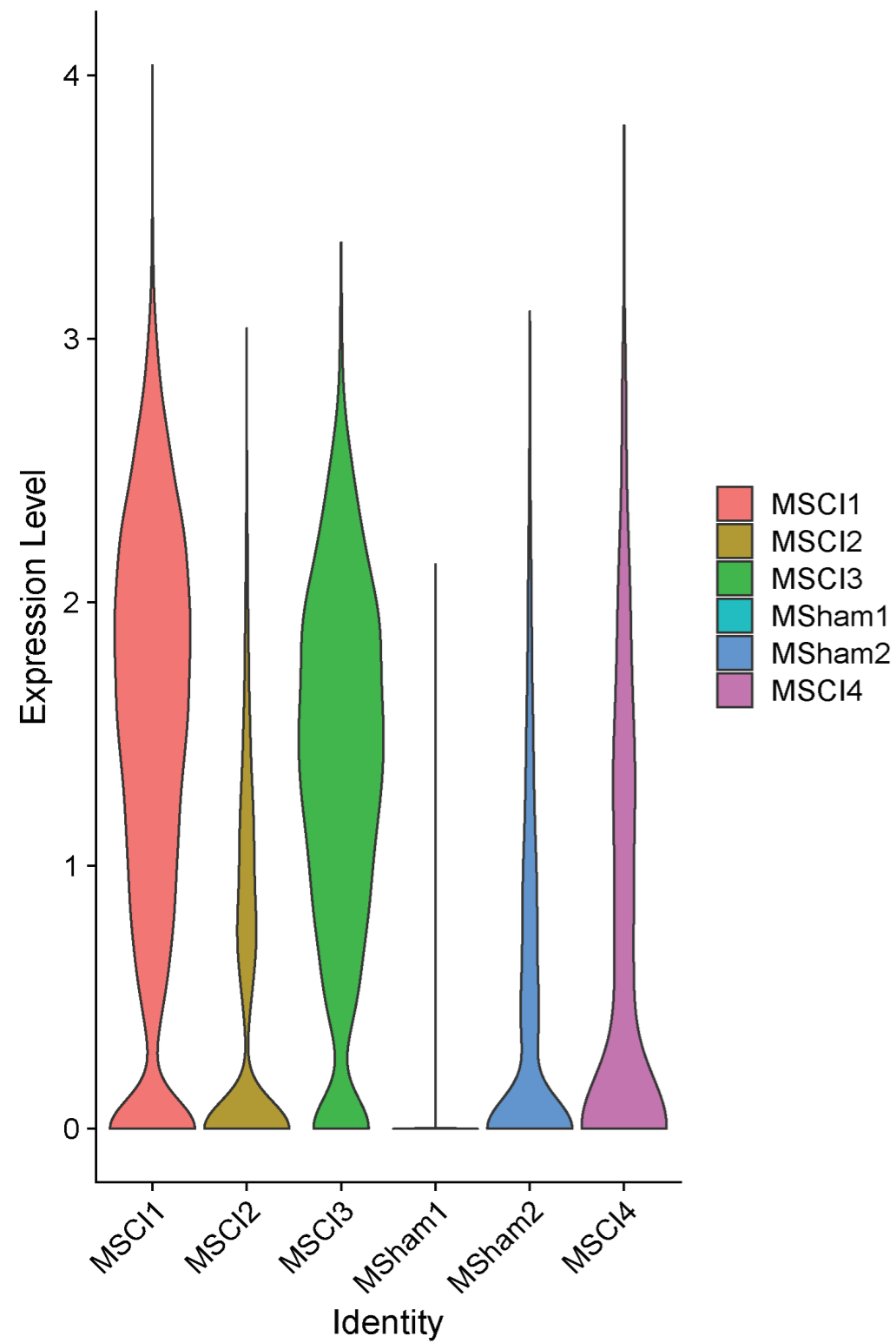**Gngt2**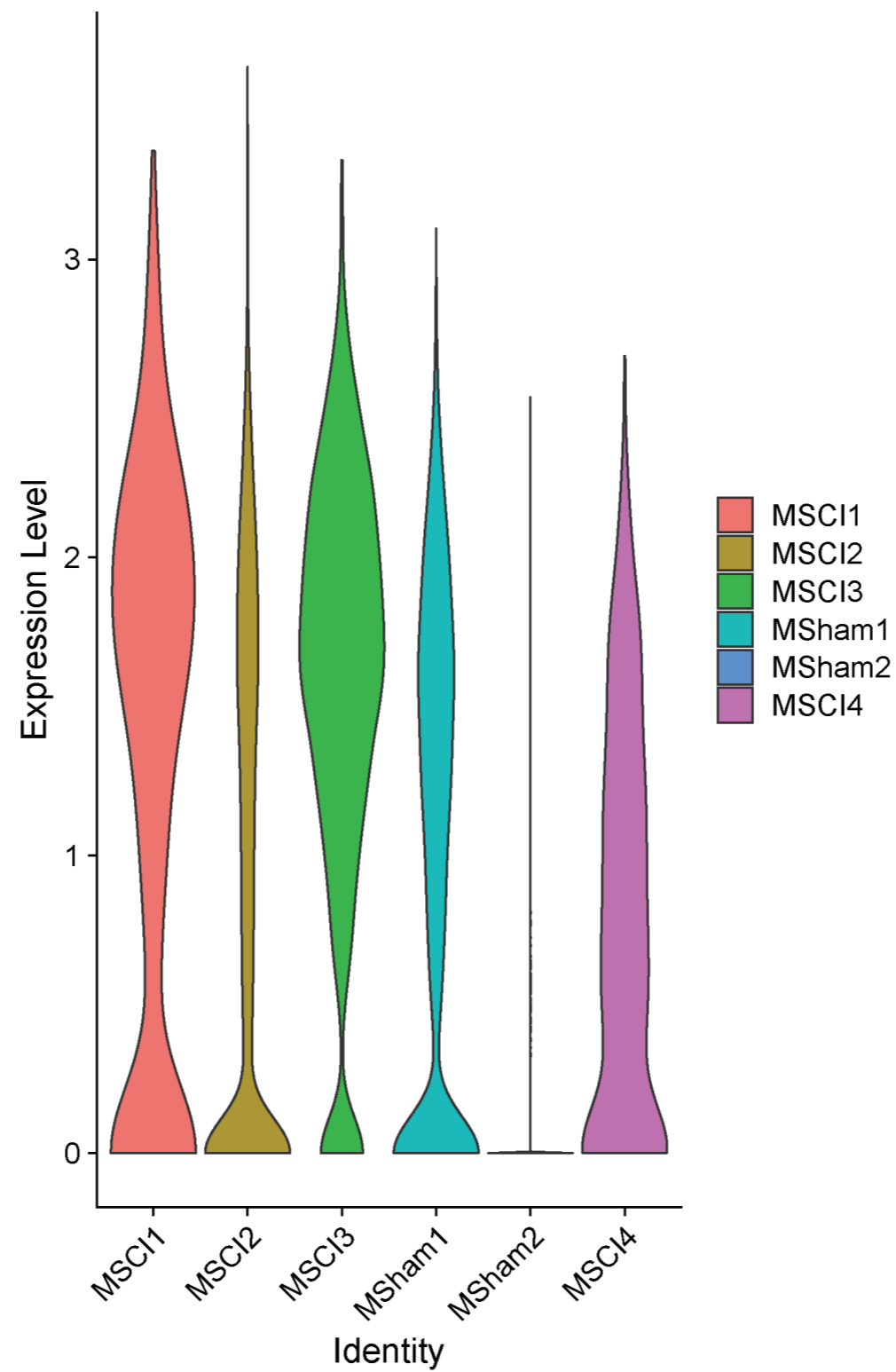**Sgpl1**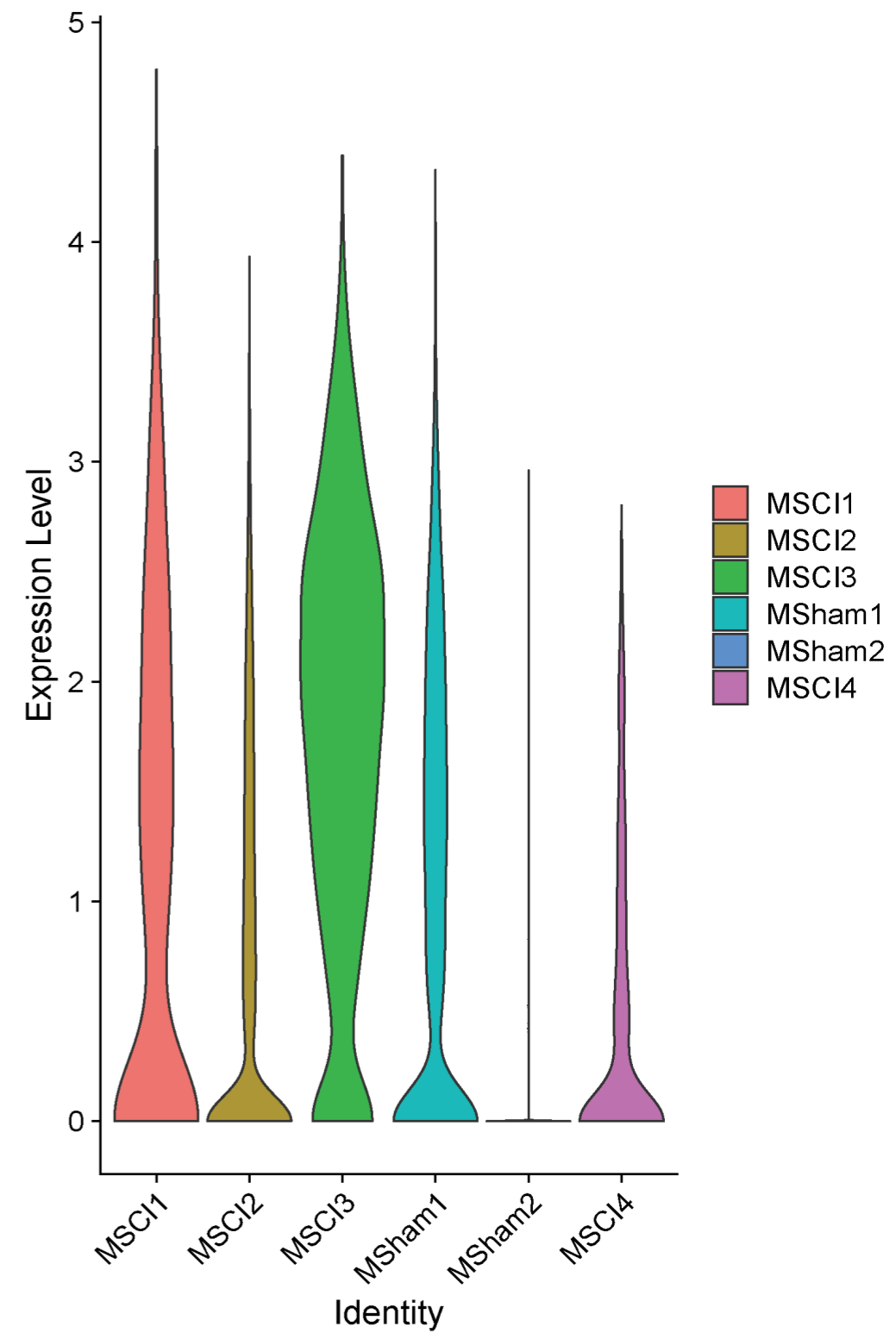

Supplement: Supplementary Figure 2 — The expression levels of the selected genes (Emp3, Gngt2, and Sgpl1) in six subpopulations of MM cells. [file DataSheet2.pdf]
